# Molecular Cloning, Expression Pattern and Genotypic Effects on Glucoraphanin Biosynthetic Related Genes in Chinese Kale (*Brassica oleracea* var. *alboglabra* Bailey)

**DOI:** 10.3390/molecules201119688

**Published:** 2015-11-11

**Authors:** Ling Yin, Changming Chen, Guoju Chen, Bihao Cao, Jianjun Lei

**Affiliations:** Department of Hortscience, South China Agricultural University, Guangzhou 510642, China; 1987.yinling@163.com (L.Y.); cmchen@scau.edu.cn (C.C.); gjchen@scau.edu.cn (G.C.); caobh01@scau.edu.cn (B.C.)

**Keywords:** Chinese kale, glucoraphanin, *BCAT4*, *MAM1*, *CYP79F1*

## Abstract

Glucoraphanin is a plant secondary metabolite that is involved in plant defense and imparts health-promoting properties to cruciferous vegetables. In this study, three genes involved in glucoraphanin metabolism, branched-chain aminotransferase 4 (*BCAT4*), methylthioalkylmalate synthase 1 (*MAM1*) and dihomomethionine N-hydroxylase (*CYP79F1*), were cloned from Chinese kale (*Brassica oleracea* var. *alboglabra* Bailey). Sequence homology and phylogenetic analysis identified these genes and confirmed the evolutionary status of Chinese kale. The transcript levels of *BCAT4*, *MAM1* and *CYP79F1* were higher in cotyledon, leaf and stem compared with flower and silique. *BCAT4*, *MAM1* and *CYP79F1* were expressed throughout leaf development with lower transcript levels during the younger stages. Glucoraphanin content varied extensively among different varieties, which ranged from 0.25 to 2.73 µmol·g^−1^ DW (dry weight). Expression levels of *BCAT4* and *MAM1* were high at vegetative–reproductive transition phase, while *CYP79F1* was expressed high at reproductive phase. *BCAT4*, *MAM1* and *CYP79F1* were expressed significantly high in genotypes with high glucoraphanin content. All the results provided a better understanding of the roles of *BCAT4*, *MAM1* and *CYP79F1* in the glucoraphanin biosynthesis of Chinese kale.

## 1. Introduction

Glucosinolates (GSLs or β-thioglucoside-*N*-hydroxysulfates) comprise a large family of over 130 nitrogen- and sulfur-rich plant amino acid-derived secondary metabolites found in the *Brassicaceae*, *Capparidaceae* and *Caricaceae* families, commonly known as cabbage, caper and papaya, respectively [1,2]. GSLs encompass three major chemical classes, aliphatic, aromatic and indole GSLs, based on their precursor amino acids and R group modifications [3]. GSLs and their hydrolysis products have important biological activities such as flavor precursors, crop protectants and cancer-prevention agents [4]. More specifically, some hydrolysis products are known to contribute to the special flavors and odors of *Brassicaceae* [5]. GSLs also play important roles in frequently-studied plant defense systems against insect, fungi, pathogens and microbial infections [6].

Of all the GSLs, glucoraphanin (4-methylsulfinylbutyl GSL), the GSL precursor of the bioactive isothiocyanate sulforaphane, is most well-known [7]. Glucoraphanin, an aliphatic GSL, is found in cruciferous vegetables such as broccoli, cabbage, cauliflower, kale and turnip. Glucoraphanin has been shown to decrease hypertension, inflammation and oxidative stress in the cardiovascular system of rats [8]. Sulforaphane (4-methylsulphinylbutyl isothiocyanate), the cognate isothiocyanate of glucoraphanin, is of prominent importance to the protective effects of cruciferous vegetables [9]. Sulforaphane has proven to be a potent inducer of mammalian phase II detoxification and antioxidant enzymes, protecting cells against carcinogens and toxic electrophiles [10].

**Figure 1:**
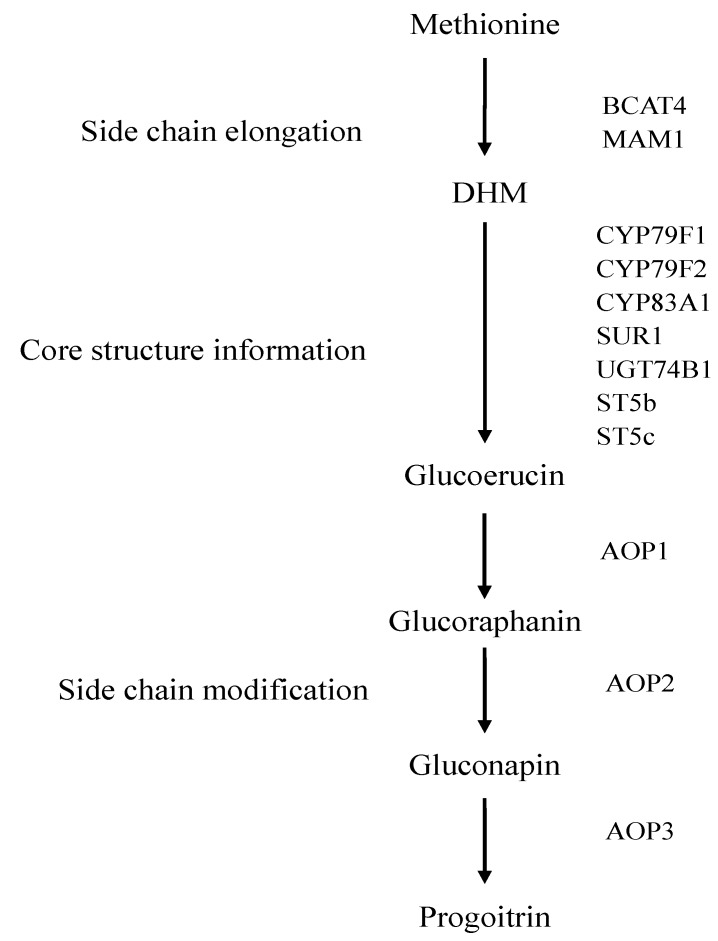
The biosynthetic pathway of glucoraphanin in *Brassica* vegetables.

The biosynthetic pathway of glucoraphanin has been basically elucidated in *Brassica* vegetables (Figure 1). The biosynthetic pathways of glucoraphanin include side-chain elongation, core structure formation and secondary modification [3,11]. Many representative genes involved in glucoraphanin metabolism and regulation have been identified in *Arabidopsis thaliana* [12,13]. Branched-chain aminotransferase 4 (*BCAT4*), methylthioalkylmalate synthase 1 (*MAM1*) have been characterized in the side-chain elongation of methionine with two methylene groups to form dihomo-methionine (DHM) [12,14,15,16]. The pathway for core structure formation is mediated by cytochromes P450 mono-oxygenases *CYP79F1*, *CYP79F2* and *CYP83A1*. The compounds are further metabolized to 4-Methylthiobutyl glucosinolate (Glucoerucin) by C-S lyases (*SUR1*), UDP-glucuronyl transferase (*UGT74B1*) and sulfotransferase (*ST5b* and *ST5c*) [17,18,19,20,21,22]. The amino acid side chain is modificated by GSL-OX (*AOP1*), GSL-ALK (*AOP2*) and GSL-OH (*AOP3*) in the side chain modification reaction [23,24]. Glucoerucin is first oxidized by *AOP1* to 4-methylsulphinylbutyl glucosinolate (glucoraphanin). Glucoraphanin is subsequently converted into 3-butenyl glucosinolate (gluconapin) by *AOP2*. Gluconapin is hydroxylated by *AOP3* to form 2-hydroxy-3-butenyl (progoitrin).

The presence of glucoraphanin in *Arabidopsis thaliana* has enabled identification of the majority of genes and intermediates involved in glucoraphanin metabolism [25,26,27]. Although the regulation of glucoraphanin metabolism is of great importance for nutrition and quality in Chinese kale, studies at the molecular level have been hampered by a lack of candidate genes. Our study focuses on the structural genes (*BCAT4*, *MAM1* and *CYP79F1*) involved in glucoraphanin metabolism. *BCAT4* is confirmed to participate in the variation in the side chain length of methionine during the synthesis of aliphatic GSLs, which has a close evolutionary relationship with the synthesis of leucine [15]. *MAM1* is known to condense acetyl-CoA with 2-oxo-4-methylthiobutanoic acid (OMTB) to form 2-(2-methylthio) ethylmalic acid (MTEM) [28]. *MAM1* can also catalyze the condensations of the first three side-chain elongation cycles [14]. *CYP79F1*, a member of the CYP79 family and a target gene in the present study, is the first enzyme to form aldoxime during the biosynthesis of aliphatic GSLs [29]. *CYP79F1* has been shown to specifically catalyze the conversion of chain-elongated methionine homologues to their corresponding aldoximes [17].

Chinese kale (*Brassica oleracea* var. *alboglabra* Bailey) belongs to the *Brassicaceae* family. Chinese kale is a rich source of antioxidants and anticarcinogenic compounds, including vitamin C, GSLs, carotenoids and phenolic compound [30]. Due to high nutritional value, the market demand for Chinese kale is increasing. Chinese kale is widely distributed in southern China, Taiwan, Japan and Southeast Asia and has spread quickly in Europe and America [31,32]. The most common edible part of Chinese kale is bolting stem, which is tender and crispy, with good flavor. In recent years, tender rosette leaves and sprouts are also consumed as leafy vegetables in southern China [23,24]. Genotype has a significant effect on glucoraphanin level in Chinese kale. Qian *et al.* [23] measured glucoraphanin content of fourteen Chinese kale cultivars bolting stems and found that the glucoraphanin content ranged from 0.33 to 2.89 µmol·g^−1^ DW (dry weight), with an average value of 1.07 µmol·g^−1^ DW. Sun *et al.* [24] measured the glucoraphanin content in 27 broccoli cultivars commonly grown in South China. The glucoraphanin content ranged from 0.14 to 1.65 µmol·g^−1^ DW, with an average value of 0.66 µmol·g^−1^ DW.

The full CDS of *BCAT4*, *MAM1* and *CYP79F1* were cloned from Chinese kale for the first time. We predicted protein sequences and performed structural and phylogenetic analysis. The expression levels of *BCAT4*, *MAM1* and *CYP79F1* were examined to determine the differences in gene expression at different tissues and stages. The content of glucoraphanin was evaluated in bolting stems of eight Chinese kale varieties. The relationship between the glucoraphanin content and the transcript levels of biosynthetic genes were discussed. The research will provide the basis for further studies on *BCAT4*, *MAM1* and *CYP79F1* and their function in the molecular mechanisms of glucoraphanin biosynthesis in Chinese kale, which will help breeders to select cultivars with high glucoraphanin content and will also provide guidance for human consumption.

## 2. Results and Discussion

### 2.1. Molecular Cloning of BCAT4, MAM1 and CYP79F1

*BCAT4*, *MAM1* and *CYP79F1*, which are essential for glucoraphanin biosynthesis, were cloned for the first time in Chinese kale. Sequence analysis of *BCAT4* indicated that the full length cDNA was 1394 bp including 42 bp 5′-untranslated region, 1143 bp open reading frame (ORF) and 209 bp 3'-untranslated region (Supplementary Figure S1a). The nucleotides encoded 380 amino acids with a predicted molecular mass of 42 kD and isoelectric point of 9.09. Searching against the Conserved Domain Database (CDD) revealed that BCAT4 contained BCAT beta family domain (position 78–358) and belonged to the PLPDE IV superfamily. The predicted secondary structure stated that BCAT4 contained 27.11% alpha helices, 37.89% random coils, 22.63% extended strand and 12.37% beta turn (Supplementary Figure S2a). The tertiary structure of BCAT4 was predicted by SWISS MODEL (Supplementary Figure S3a).

The full-length cDNA sequence of *MAM1* was composed of 1560 bp (Supplementary Figure S1b). The cDNA sequence contained an 1164 bp open reading frame that encoded an estimated polypeptide of 387 amino acids with a molecular weight of 42 kDa and an isoelectric point of 5.99. The full length MAM1 cDNA also consisted of 291 bp 5′-untranslated region and 105 bp 3′-untranslated region. MAM1 contained DRE TIM IPMS domain (position 1–245) and belonged to TIM phosphate binding superfamily as determined by comparison with the CDD. Secondary structure prediction by SDPMA showed that MAM1 contained 37.98% alpha helices, 31.27% random coils, 19.38% extended strand and 11.37% beta turn (Supplementary Figure S2b). The 3-D structural modeling of MAMl was performed online with SWISS MODEL (Supplementary Figure S3b).

Analysis of the nucleotide sequence indicated that the cDNA of *CYP79F1* was 1685 bp with an ORF of 1626 bp (Supplementary Figure S1c). The full-length cDNA comprised 27 bp 5'-untranslated region and 32 bp 3'-untranslated region. The deduced CYP79F1 protein was a polypeptide of 541 amino acid with a putative molecular mass of 61 kDa and a pI of 8.26. Domain architecture analysis suggested that CYP79F1 contained p450 domain (position 49–522) and belonged to the p450 superfamily. The secondary structure of CYP79F1 was predicted to be 41.77% alpha helices, 35.12% random coils, 15.71% extended strand and 7.39% beta turn (Supplementary Figure S2c). A similar result was obtained with CYP79F1 three-dimensional structure constructed using SWISS MODEL software (Supplementary Figure S3c).

### 2.2. Bioinformatics Analysis of BCAT4, MAM1 and CYP79F1

Bioinformatics was used to predict the structure and function of *BCAT4*, *MAM1* and *CYP79F1*. The transmembrane domains and cell functional status are closely related [33,34]. The accuracy of the predicted transmembrane region is not more than 52% by software [35]. The transmembrane helix may be not predicted by two methods, exact numbers need to be validated by experiments.

The TMHMM result indicated that BCAT4 had no obvious transmembrane domain, which implied that BCAT4 is neither a membranous acceptor nor can be located in the membrane. The TMpred analysis predicted that BCAT4 consisted of three possible transmembrane helices. No signal peptide was identified by the SignalP 4.1. No *N*-glycosylation site was identified in BCAT4. Phosphorylation site prediction revealed that ten serine, seven threonine and six tyrosine targets existed in BCAT4. *In silico* analysis predicted nine potential *O*-glycosylation sites in BCAT4 using the program YinOYang. We examined the hydrophobic/hydrophilic nature of BCAT4 using ProtScale software and found a maximum value of 2.2 and a minimum value of −2.644. Hydrophilic amino acid residues predominate in the peptide chain, indicating that BCAT4 might be a hydrophilic protein.

The TMHMM result revealed that MAM1 had no obvious transmembrane domain. The TMpred analysis revealed that MAM1 had one possible transmembrane helix. SignalP software analysis revealed that no signal peptide was identified in MAM1. A single *N*-glycosylation site was identified in position 365. The phosphorylation sites were predicted to be as follows: twelve serine, five threonine and seven tyrosine. YinOYang analysis revealed that five potential *O*-glycosylation sites exist in MAM1. ProtScale software analysis revealed that the maximum value was 2.589 and the minimum value was −2.733, indicating that MAM1 might be a hydrophilic protein.

The TMHMM result indicated that CYP79F1 contained a transmembrane domain at residues 13–35, which implied that CYP79F1 may be a membranous acceptor or ion channel protein and may be located in the membrane. The TMpred analysis indicated that CYP79F1 had two possible transmembrane helices. No signal peptide was identified by the SignalP software. NetNGlyc analysis revealed that a single *N*-glycosylation site was identified in position 43. Eleven serine, six threonine and three tyrosine targets were predicted in CYP79F1. Two *O*-glycosylation sites were identified in CYP79F1. We found a maximum value of 2.644 and a minimum value of −2.9 using ProtScale software, indicating that CYP79F1 might be a hydrophilic protein.

### 2.3. Phylogenetic Analysis of BCAT4, MAM1 and CYP79F1

The evolutionary relationship and classification of species could be approximated based on phylogenetic analysis [36,37]. In order to evaluate the molecular evolutionary relationships, neighbor-joining method was used to construct phylogenetic trees of BCAT4 (Supplementary Figure S4), MAM1 (Supplementary Figure S5) and CYP79F1 (Supplementary Figure S6). The phylogenetic relationships were in accordance with the classification and evolutionary status via morphological and biochemical characteristics. The distances between different genera were relatively large, while the distances within the same genus were relatively small. Multi-alignment by Clustal X2 showed that BCAT4, MAM1 and CYP79F1 in Chinese kale were phylogenetically closer to *Brassica* plants, including *Brassica napus*, *Brassica rapa*, *Brassica oleracea*, *Brassica juncea*, *Brassica rapa* subsp. *rapa*, *Brassica oleracea* var. *alboglabra*, *Brassica rapa* subsp. *pekinensis* and *Brassica rapa* subsp. *chinensis*.

### 2.4. Spatial and Temporal Expression Patterns of BCAT4, MAM1 and CYP79F1

The expression patterns analysis of genes is useful to understand their physiological roles. Several genes involved in glucoraphanin metabolism showed differential expression patterns in different kinds of species including *Arabidopsis thaliana*, *Brassica napus*, *Raphanus sativus* and *Armoracia rusticana* [38,39,40,41]. *BCAT4* has been found to catalyze the initial reaction of the Met chain elongation pathway [15,25]. In *Arabidopsis thaliana*, cytoplasmic *BCAT4*, is mainly expressed in tissues associated with transport functions, such as the phloem cells in the direct vicinity of S-cells [38]. *MAM1* controls C3/C4 chain length variation and is considered an essential gene for the biosynthesis of Met-derived glucosinolates [14,42]. Contrary to BCAT4, MAM1 is found exclusively in the phloem. The fact that BCAT4 and MAM1 are transcribed in the same tissues suggests that the transcription of *BCAT4* is closely correlated with that of *MAM1*. *CYP79F1* catalyzes the conversion of dihomomethionine and trihomomethionine to the corresponding aldoximes in the biosynthesis of aliphatic glucosinolates [17,43]. The expression level of *CYP79F1* in the rosette leaves was higher than that in the petioles of *Arabidopsis thaliana* [17].

**Figure 2:**
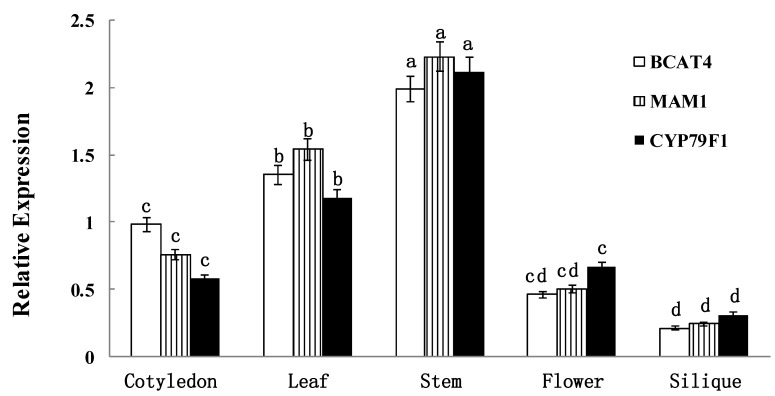
Spatial expression patterns of *BCAT4*, *MAM1* and *CYP79F1*. The tissues are defied as: cotyledon (7 days), leaf (15 days), stem (30 days), flower (anthesis) and silique (15 days post-anthesis). Each bar represents the mean (±standard error) of three independent biological replicates. Different letters on the top indicate significant differences at *p* < 0.05.

To the best of our knowledge, no studies have reported on spatial and temporal expression levels of *BCAT4*, *MAM1* and *CYP79F1* in Chinese kale. This research was the first on this subject. qRT-PCR analysis revealed that all three genes were expressed in most of the tissue types (Figure 2). The expression patterns of *BCAT4*, *MAM1* and *CYP79F1* in different tissues were associated with the biological activities and simultaneous functions. *BCAT4*, *MAM1* and *CYP79F1* were constitutively expressed in all of the collected tissues, under normal growth conditions, in Chinese kale. The expression patterns of *BCAT4*, *MAM1* and *CYP79F1* in different tissues were associated with the biological activities and simultaneous functions. *BCAT4*, *MAM1* and *CYP79F1* were also tissue-specific. All three genes were highly expressed in glucosinolate synthesizing tissues such as cotyledon, leaf and stem, whereas flower and silique showed week transcript levels. Higher expression levels of *BCAT4* and *MAM1* were observed in cotyledon, leaf and stem compared with flower and silique. *CYP79F1* was highly expressed across all the developing stages, particularly in leaf and stem. The high expression levels in cotyledon, leaf and stem indicate that *BCAT4*, *MAM1* and *CYP79F1* also play important roles in those tissues.

**Figure 3:**
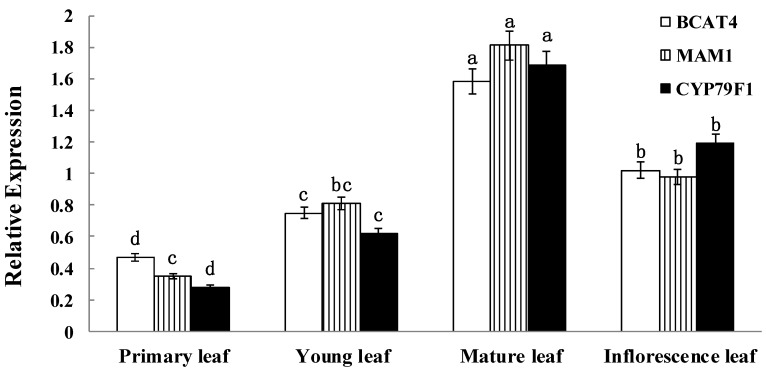
Temporal expression patterns of *BCAT4*, *MAM1* and *CYP79F1*. The stages are defied as: primary leaf (15 days), young leaf (30 days), mature leaf (60 days) and inflorescence leaf (anthesis). Each bar represents the mean (±standard error) of three independent biological replicates. Different letters on the top indicate significant differences at *p* < 0.05.

Detailed expression analysis of *BCAT4*, *MAM1* and *CYP79F1* were further performed at different developmental stages of leaves in Chinese kale. quantitative real-time PCR (qRT-PCR) analysis showed significant differences in the expression of all three genes among the different developmental stages (Figure 3). The data clearly reflect the temporal expression of *BCAT4*, *MAM1* and *CYP79F1*. Expression patterns of *BCAT4*, *MAM1* and *CYP79F1* in different stages also provide a basis for further exploring their physiological role in mediating glucoraphanin metabolism of Chinese kale. *BCAT4*, *MAM1* and *CYP79F1* had relatively high transcript accumulation in the mature and inflorescence leaves with the onset of the reproductive phase. During the developing stages of the leaves, expression levels of *BCAT4* and *MAM1* were comparably higher than *CYP79F1*.

### 2.5. Glucosinolate Contents in Different Varieties of Chinese Kale

The most important factor determining glucoraphanin content has been reported to be genotype [34,44,45]. In the present study, the glucosinolates contents in bolting stems of eight Chinese kale cultivars were determined by high performance liquid chromatography (Table 1). The results revealed that significant differences in the glucosinolates contents occurred among different varieties.

**Table 1:** Glucosinolate composition and content (µmol·g^−1^ DW) in Chinese kale bolting stems of different varieties.

	Glucoerucin	Glucoraphanin	Gluconapin	Total Glucosinolates
Bo-1	1.35 b	2.73 a	9.07 a	15.12 a
Bo-2	1.02 c	2.23 b	8.93 a	14.17 a
Bo-3	1.62 a	1.53 c	7.16 b	11.12 b
Bo-4	0.72 cd	1.62 c	5.89 bc	9.85 c
Bo-5	0.47 e	0.92 d	7.45 b	10.75 bc
Bo-6	0.76 cd	1.03 d	3.28 d	5.11 f
Bo-7	0.64 d	0.58 e	5.04 c	7.02 d
Bo-8	0.21 f	0.25 f	3.55 d	5.75 e

Each value represents the mean (*n* = 3). Values in the same column followed by the same letter are not significantly different at *p* < 0.05.

The glucoraphanin content ranged from 0.25 to 2.73 µmol·g^−1^ DW, with an average value of 1.36 µmol·g^−1^ DW. The highest content of glucoraphanin was found in Bo-1, while the lowest content of glucoraphanin was detected in Bo-8. Genetic variation provides the potential to produce new varieties of Chinese kale with optimal glucoraphanin content. The differences in glucoraphanin content among varieties in Chinese kale were in coincidence with previous observations [23,24].

The level of glucoerucin, the direct precursor of glucoraphanin, ranged from 0.21 to 1.62 µmol·g^−1^ DW, with an average value of 0.85 µmol·g^−1^ DW. The lowest content of glucoerucin was observed in Bo-8, while the highest content of glucoerucin was detected in Bo-3. The level of gluconapin, the alkenyl product of glucoraphanin, ranged from 3.28 to 9.07 µmol·g^−1^ DW, with an average value of 6.3 µmol·g^−1^ DW. The stems of Bo-1 had the highest content of gluconapin, while the stems of Bo-6 had the lowest content of gluconapin.

The level of total glucosinolate ranged from 5.11 to 15.12 µmol·g^−1^ DW, with an average value of 9.86 µmol·g^−1^ DW. The lowest content of total glucosinolate was observed in Bo-6, while the highest content of total glucosinolate was detected in Bo-1. In terms of the total glucosinolate levels, the proportion of gluconapin was 60%–72%, while glucoraphanin and glucoerucin were 4%–20%, Qian *et al.* [23] demonstrated that gluconapin was the most abundant aliphatic glucosinolate, which represented over 60% of the total glucosinolate content in most varieties of Chinese kale. The percentage of glucoraphanin was lower than 25% of the total glucosinolate content. Similar results were also reported in several other *Brassica* species, such as turnip greens and Chinese cabbage [46,47]. Most varieties of turnip greens had a proportion of gluconapin over 70% of the total glucosinolate content and a proportion of glucoerucin below 20% of the total glucosinolate content. Glucoraphanin was also presented in 27 of 113 varieties of turnip greens [46]. In Chinese cabbage, the proportion of gluconapin was 65%–75%, while glucoraphanin and glucoerucin were less than 7% of total glucosinolate [47].

### 2.6. Genotypic Expression Patterns of BCAT4, MAM1 and CYP79F1

**Figure 4:**
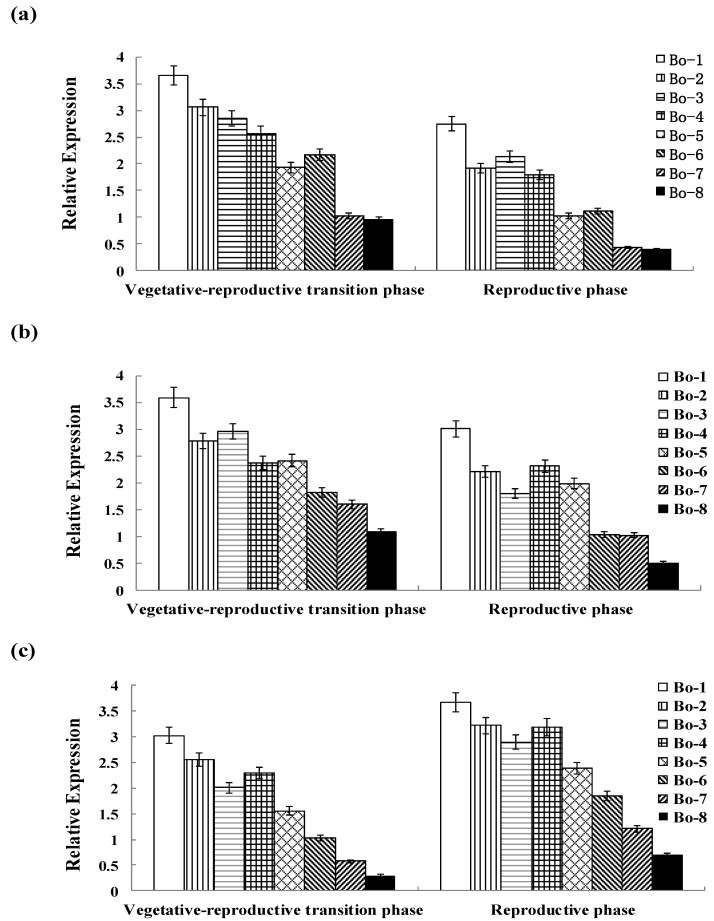
(**a**) Genotypic expression patterns of *BCAT4*; (**b**) Genotypic expression patterns of *MAM1*; (**c**) Genotypic expression patterns of *CYP79F1*. The stages are defied as: vegetative–reproductive transition phase (the elongated stems) and reproductive phase (stems with inflorescence). Each bar represents the mean (±standard error) of three independent biological replicates.

Differential expression of functional genes may reflect differences in the efficiency of material transportation and absorption [48]. The bolting stems from eight varieties were selected as test materials for further analysis of gene expression based on the differences in the glucoraphanin content. In order to better understand the expression patterns of *BCAT4*, *MAM1* and *CYP79F1*, qRT-PCR analysis was used to validate the dynamic expression patterns of the three glucoraphanin biosynthesis related genes at two developmental stages. The results revealed that *BCAT4*, *MAM1* and *CYP79F1* exhibited different expression patterns among different stages (Figure 4). Expression of all three genes appears to be directly associated with the glucoraphanin content in different Chinese kales. In addition, the contents of glucoerucin and gluconapin, the direct precursor and alkenyl product of glucoraphanin, had a relationship with expression levels of *BCAT4*, *MAM1* and *CYP79F1.*

There might be a positive correlation between gene expression and glucoraphanin content. The expression levels of *BCAT4* and *MAM1* were higher at vegetative–reproductive transition phase than those at reproductive phase. This down-regulation of gene expression might be correlated with the decrease in the glucoraphanin content. In contrast, the expression of *CYP79F1* was significantly up-regulated from vegetative-reproductive transition phase to reproductive phase. There was a significant increase of 1.2–1.6-fold in the transcript of *CYP79F1* at reproductive phase in eight genotypes. *CYP79F1* was expressed at higher level during the reproductive phase in contrast to *BCAT4* and *MAM1*, which indicated that *CYP79F1* played an important role in the late stage of glucoraphanin metabolism. The higher expression levels of *BCAT4*, *MAM1* and *CYP79F1* were found to be in accordance with the higher glucoraphanin concentration of Chinese kale. The expression levels of the analyzed genes were significantly higher in genotypes with high glucoraphanin content than those with low glucoraphanin content.

## 3. Experimental Section

### 3.1. Plant Materials and Growth Conditions

Eight local varieties of Chinese kale were collected from South China. The seeds of Chinese kale were germinated in plastic pots and the seedlings were cultured in the field at 22–25 °C at South China Agricultural University (Guangzhou, China). Field managements were the same as general production. Water, pesticides and fertilizer were applied as necessary. Bolting stems (free of any insects and mechanical damage) were harvested when plants were fully grown, with inflorescence as high as the apical leaves for cDNA sequence cloning.

For spatial expression patterns analysis, cotyledon (7 days), leaf (15 days), stem (30 days), flower (anthesis) and silique (15 days post-anthesis) were excised from several plants. For temporal expression patterns analysis, primary leaf (15 days), young leaf (30 days), mature leaf (60 days) and inflorescence leaf (anthesis) were excised from several plants. For genotypic expression patterns analysis, the stems of different varieties were collected at two stages including vegetative-reproductive transition phase (the elongated stems) and reproductive phase (stems with inflorescence). Three independent replicates were taken for each sample analysis.

Plant materials were harvested in early morning, weighed fresh and washed with distilled water. All samples were immediately transported to the laboratory within 10 min, frozen in liquid nitrogen, and lyophilized in an ultralow −80 °C freezer to determine the ratio of fresh weight (FW) to dry weight (DW). The lyophilized samples were ground into fine powders and stored at −20 °C for further analysis of glucoraphanin content.

### 3.2. RNA Extraction and cDNA Synthesis

Total RNA of Chinese kale was isolated using TRIzol reagent (Invitrogen, Carlsbad, CA, USA) following the manufacturer’s instructions. During extraction procedures, total RNA was exhaustively treated with RNase-free Dnase (Qiangen, Germany) to remove possible DNA contamination. RNA quality and content were determined with gel electrophoresis containing 1.2% agarose gel and biophotometer (Eppendorf, Germany). The first-strand cDNA was synthesized from 1 μg total RNA using a Reverse Transcriptase M-MLV Kit (Takara, Japan).

### 3.3. Molecular Cloning of BCAT4, MAM1 and CYP79F1

Degenerate primers were designed according to *BCAT4*, *MAM1* and *CYP79F1* sequences identified at the BRAD *Brassica* database [49] using Primer 5.0 (Supplementary Table S1). PCR was carried out in a total volume of 25 μL. The PCR program was performed as follows: activation for 5 min at 95 °C, 35 cycles of denaturation for 1 min at 95 °C, extension for 1 min at 58 °C and termination for 1 min at 72 °C, with the final extension step at 72 °C for 10 min. PCR products were isolated and ligated into the pMD19-T vector (Takara, Japan), prior to transformation in *Escherichia coli* (*E. coli*) DH5α. The three sequences were submitted to GenBank and the corresponding accession numbers were KP295464, KP295465 and KP295466 for *BCAT4*, *MAM1* and *CYP79F1*, respectively.

### 3.4. Expression Analysis of BCAT4, MAM1 and CYP79F1

The expression patterns of the target genes were assayed by quantitative real-time PCR (qRT-PCR). Three pairs of primers (Supplementary Table S1) were designed based on *BCAT4*, *MAM1* and *CYP79F1* sequences to amplify the fragment of 247 bp, 269 bp and 249 bp, respectively.

The reaction was performed using the SYBR Green Master (Roche, Swiss) on MyiQ Real-Time PCR Detection System (Bio-Rad) platform in a total 20 μL volume according to manufacturer’s protocols. The relative expression levels of the reference genes were calculated using the 2^−ΔΔCt^ method.

The amplification protocol was as follows: denaturation for 2 min at 95 °C, 40 cycles of 15 s at 95 °C, 30 s at 58 °C and 30 s at 72 °C, with a 5 min final extension at 72 °C. Actin was selected as the internal control gene.

All PCR reactions were performed in triplicate as technical replicates. Three independent biological duplications were also carried out for all target genes. Reverse transcription negative and non-template controls were run to detect potential genomic DNA and reagent contamination, respectively. Data were analyzed using Bio-Rad CFX Manager software.

### 3.5. Extraction and Determination of Glucosinolates

Glucosinolates were extracted and analyzed as previously described [50]. The freeze-dried samples (500 mg) were mixed with 2 mL of 75% methanol at 80 °C for 15 min. The samples were cooled to room temperature and centrifuged at 10,000× *g* for 5 min. The supernatant was transferred to a new tube after centrifugation. The residues were washed with 2 mL of 75% methanol, centrifuged at 10,000× *g* for 5 min and combined with the previous supernatant. The combined supernatant was applied onto DEAE-Sephadex A-25 column (acetic acid activated) and rinsed three times with 2 mL 0.02 mol/L sodium acetate. The column was washed three times with 20 mM acetic acid and twice with water. The supernatant was mixed with 200 μL arylsulfatase solution. After incubation for 16 h at 35 °C, the desulphoglucosinolates were eluted with 4 mL of Milli-Q water and filtered through a 0.45 μm membrane filter. High performance liquid chromatography (HPLC) analysis of desulphoglucosinolates were carried out as previously described [32]. HPLC analysis was performed by Waters HPLC instrument equipped with Model 2996 PDA absorbance detector (Waters, Milford, MA, USA). Samples (20 μL) were separated on a Waters Spherisorb C18 column (250 mm × 4.6 mm, 5 μm) at a flow rate of 1.0 mL·min^−1^. Compounds were separated utilizing the following program: gradient 1.5% acetonitrile for the first 5 min, 15 min linear gradient from 1.5% to 20%, gradient 20% acetonitrile for the final 10 min. Absorbance of desulphoglucosinolates were quantified by the A226 nm. Data were extracted using *Ortho*-nitrophenyl-β-d-galactopyranoside (Sigma, Steinheim, Germany) as an internal standard.

### 3.6. Bioinformatic Analysis

Amino acid sequences of *BCAT4*, *MAM1* and *CYP79F1* were deduced using DNAman software. The amino acid sequences were aligned using ClustalW (http://www.genome.jp/tools/clustalw/). The phylogenetic trees were generated based on the NJ (neighbor joining) sequences distance method and constructed using MEGA 6.06 (http://www.megasoftware.net/). The Bootstrap method was used to derive the confidence value for the phylogeny analysis. Conserved Domain prediction was performed by CDD (http://www.ncbi.nlm.nih.gov/Structure/cdd/cdd.shtml). Molecular weight and isoelectric point were calculated using the ProtParam tool (http://web.expasy.org/protparam). The secondary structures were predicted by SOPMA (https://npsa-prabi.ibcp.fr/cgi-bin/npsa_automat.pl?page=npsa_sopma.html). The tertiary structures were predicted by SWISS MODEL (http://swissmodel.expasy.org). The transmembrane domains of the amino acid sequences were predicted by TMHMM 2.0 Server (http://www.cbs.dtu.dk/services/TMHMM/). Topology was determined using TMpred (http://www.ch.embnet.org/software/TMPRED_form.html). The signal peptides were predicted using SignalP 4.1 Server (http://www.cbs.dtu.dk/services/SignalP/). The putative *N*-glycosylation sites of the amino acid sequences were predicted by NetNGlyc 1.0 Server (http://www.cbs.dtu.dk/services/NetNGlyc/). The putative phosphorylation sites were predicted using the program NetPhos 2.0 (http://www.cbs.dtu.dk/services/NetPhos/). Putative *O*-glycosylation sites were predicted by the program YinOYang 1.2 (http://www.cbs.dtu.dk/services/YinOYang/). The hydrophilic and hydrophobic regions were predicted using ProtScale (http://web.expasy.org/protscale/).

### 3.7. Statistical Analysis

The SPSS package program version 11.5 (SPSS Inc. Chicago, IL, USA) was used for statistical analysis. Data were analyzed by one-way ANOVA model. The means were determined by the Least Significant Differences (LSD) test at *p* = 0.05.

## 4. Conclusions

In conclusion, full length cDNAs of BCAT4, MAM1, and CYP79F1 were isolated for the first time in Chinese kale, with the goal of facilitating further studies on the regulation of glucoraphanin metabolism at the molecular level. The sequence homology and phylogenetic relationship were analyzed. The expression patterns were detected, which provided a basis for further exploring their physiological role in mediating glucoraphanin metabolism. The glucoraphanin content ranged from 0.25 to 2.73 µmol·g^−1^ DW. The results obtained showed the significant correlations between the glucoraphanin content and the expression levels of *BCAT4*, *MAM1* and *CYP79F1* at vegetative-reproductive transition phase and reproductive phase. The results suggest that *BCAT4*, *MAM1* and *CYP79F1* play significant roles in Chinese kale glucoraphanin metabolism and could therefore facilitate further studies on the physiological functions and regulatory mechanisms of glucoraphanin at the molecular level. However, additional studies are required to determine the roles of *BCAT4*, *MAM1* and *CYP79F1* in other tissues such as roots during development, in order to gain further understanding of the regulation of glucoraphanin biosynthetic related genes in Chinese kale.

## Supplementary Materials

Supplementary materials can be accessed at: http://www.mdpi.com/1420-3049/20/11/19688/s1.
