# Supplementary material for: Molecular Cloning, Expression Pattern and Genotypic Effects on Glucoraphanin Biosynthetic Related Genes in Chinese Kale (Brassica oleracea var. alboglabra Bailey)
Source: Molecules. 2015 Nov 11;20(11):20254–67. doi: 10.3390/molecules201119688 (PMC6332273; doi:10.3390/molecules201119688)
Supplement: Supplementary file 1 [file molecules-20-19688-s001.pdf]

Supplementary Materials: Molecular Cloning, Expression Pattern and Genotypic Effects on Glucoraphanin Biosynthetic Related Genes in Chinese Kale (*Brassica oleracea* var. *alboglabra* Bailey)

Ling Yin, Changming Chen, Guoju Chen, Bihao Cao and Jianjun Lei

Table S1. PCR primers used for the study.

Primers	Primer Sequences (5′-3′)	Description
BCAT4-F1	TAGGATTCGGGTTTTGTCGT	Sequence cloning, forward
BCAT4-R1	AGAGCGTTTTGGTCTGGTCG	Sequence cloning, reverse
MAM1-F1	GACCGTACAATAAGCAGCCTGC	Sequence cloning, forward
MAM1-R1	TAAATCCCTGAACCGTGAGAAG	Sequence cloning, reverse
CYP79F1-F1	ATGATCCTTACCACATCGTTAC	Sequence cloning, forward
CYP79F1-F2	GTGGGTCTTGATAGTATTCGGC	Sequence cloning, reverse
BCAT4-F2	GGTCGAGGATAAGAAGGGGTG	Expression analysis, forward
BCAT4-R2	TCCAAAGGGCCAAAGGAGTAG	Expression analysis, reverse
MAM1-F2	ATAGTTGGAGCCAGCTGTTTT	Expression analysis, forward
MAM1-R2	GCATCCGTGATTCTCTTTTTA	Expression analysis, reverse
CYP79F1-F2	CATGGAATGGACACTTGCGGA	Expression analysis, forward
CYP79F1-R2	TGAATGTGGCTACCTTTGGGA	Expression analysis, reverse
Actin-F	TTGTTGGTAGGCCAAGACAT	Expression analysis, forward
Actin-R	GGAGCTCGTTGTAGAAAGTG	Expression analysis, reverse

(a)

1 CCTTTTGCTCCTTCCCTTGTCCAAAATTGGTTTTGCCCGCCAATGTGGCTTCGGCGAGGGTTTCTTACA
1 M W L R R G F L T
70 CAAAAGCAACTCGACTATCAAAACAGGAAATCCATGGCTCCTTCTGCGCGATCTCTTCCTACAAGTGTT
10 Q K Q L D Y Q N R K S M A P S A R S L P T S V
139 TCGGATGAGAAATACGCGAATGTGAAGTGGGAAGAATTAGGATTCGGGTTTTGTCGTACCGACAACATG
33 S D E K Y A N V K W E E L G F G F C R T D N M
208 TATGTTGCCAAGTGCAAACATGGAGAGAGTTTCCAAGAGGGGAAAATCGTTCCCTATGCTGATATCCAA
56 Y V A K C K H G E S F Q E G K I V P Y A D I Q
277 ATCAGCCCTTGCTCTGCAGTTCTTAACTATGGCCAGGGCTTATATGAAGGGCTTAAGGCGTACAGGACA
79 I S P C S A V L N Y G Q G L Y E G L K A Y R T
346 GAAGATGGCCGGATTGTGCTATTCCGACCAGACCAAAACGCTCTCCGCCTTCAATCGGGTGCCAACAGA
102 E D G R I V L F R P D Q N A L R L Q S G A N R
415 CTTTGTATGCCTTATCCCACGGTCGATCAATTCGTCTCCGCCGTCAAACAAGTTGTTCTTGCCAACAAG
125 L C M P Y P T V D Q F V S A V K Q V V L A N K
484 AAATGGATTCCTCCTCCGGGGAGAGGAACATTGTATATCAGACCAATCTTGTTTGGTAGTGGTCCTATA
148 K W I P P P G R G T L Y I R P I L F G S G P I
553 CTTGGCTCACTTCCTGTTCCCGAGTACACCTTCACAGTGTTTGCATGTCCCGTTGGACGTTATCACAAG
171 L G S L P V P E Y T F T V F A C P V G R Y H K
622 GATAACACGGGGTTGAACCTGAAAATTGAAGATAAGTTTCGTCGTGCTTTTCCAAGTGGAACCGGTGGT
194 D N T G L N L K I E D K F R R A F P S G T G G
691 GTTAAGAGTATCACAAACTATTCTCCTGTTTGGATAACATTAGCAGAGGCGAAAGCGCAAGGTTTCTCT
217 V K S I T N Y S P V W I T L A E A K A Q G F S
760 GATGTTTTGTTTTTGGCTGCTGCAACTGGCAAAAACGTCGAAGAGCTTTTCGCTTCTAACGTTTTCATA
240 D V L F L A A A T G K N V E E L F A S N V F I
829 GTCAAGGGAAATGTTGTGTCGACTCCAGAGATTTCAGGAACAATATTGCCCGGAGTCACACGTAAAAGT
263 V K G N V V S T P E I S G T I L P G V T R K S
898 GTCATCGAATTAACTCGTGATTTCGGCTACAAGGTTGAGGAACGTGTTGTTCCCGTTGAGGATCTTCTC
286 V I E L T R D F G Y K V E E R V V P V E D L L
967 GATGCAGAAGAAGTTTTCTGCACTGGAACTGCTGCAATTGTGACAACTATTGCGTCCGTAACCTTCAAG
309 D A E E V F C T G T A A I V T T I A S V T F K
1036 GAGAAAAAGACTGAATTCAAAACAGGTGATAAGACATTGGCTGCGAAGCTCTTTGCGACGTTAACGGAT
332 E K K T E F K T G D K T L A A K L F A T L T D
1105 ATCCAGATGGGCCGGGTCGAGGATAAGAAGGGGTGGATAGTGGAGCTGACTGATGCCACCAAACCAGGG
355 I Q M G R V E D K K G W I V E L T D A T K P G
1174 TTGAAACTTTGAACTTTATTTGAAGCTGTAACTTGACAAATTATATAAGAAACATCAGAAGATGTCTCT
378 L K L *
1243 CGATCTTTGTGTTTTATCATATTATGTCATGTTTAAGGGTCTTAAAGGGTTGTATGAAATTTATAAATA
1312 AAATTTAAGGGGTTTTTAAGTTTTACCTATCTACTACTCCTTTGGCCCTTTGGATGGAGAAGTGCGAGA
1381 AAGCGAGAGAGAGG

Figure S1. *Cont.*

(b)

```
1      CGGTCATTCTTACCCTTTGGATCTGTCCGCCTGACCCGTCCGTACAACAAGTCGTCCTTGCTCATCTCA
70     TGTTGCTCCTCTGTGTCCAAGAAGGCTGAGACTAGTGGTACTGACCTCAAAACCGTTGTGGAACGGTGG
139    CCAGAGTATATTCCGAACAAGCTCCCCGACAAGAACTACGTGCGTGTATTTGATACGACGCTCCGTGAC
208    GGCGAACAATCTCCCGGTGCAGCCCTTACTCCACCGCAGAAGATAGAGATTGCCCGGCAGCTCGCTAAA
277    CTCAGAGTAGACATCATGGAAGTTGGTTTTCCCGTGTCATCAGAGGAAGAGTTCGAAACCATCAAAACC
1                     M  E  V  G  F  P  V  S  S  E  E  E  F  E  T  I  K  T
346    ATCGCCAAAACCGTGGGAAACGAGGTTGATGAGGAAACAGGTTATATCCCAGTGATATGCGTCATCGCA
19      I  A  K  T  V  G  N  E  V  D  E  E  T  G  Y  I  P  V  I  C  V  I  A
415    CGAAGCAAAGAAAGAGATATAAAGGCGGCTTGGGAGTCAGTGAAGTACGCAAAGAGGCCGAGGATAGTC
42      R  S  K  E  R  D  I  K  A  A  W  E  S  V  K  Y  A  K  R  P  R  I  V
484    ATATTCACTTCTACTAGTGACATTCACTTGAAATATAAGTTGAAAATGACTAGAGAAGAAGTCGTCGAG
65      I  F  T  S  T  S  D  I  H  L  K  Y  K  L  K  M  T  R  E  E  V  V  E
553    ATGGTCGCGAGTAGCATTAGGTTTGCTAAAAGTTTAGGCTTCGAAGACATCGAGTTTGGTTGCGAAGAT
88      M  V  A  S  S  I  R  F  A  K  S  L  G  F  E  D  I  E  F  G  C  E  D
622    GGCGGCAGGTCCGACAAGGATTATATATGCAAGGTTTTTGAAGAAGCGATCAAAGCGGGTGCAACCACC
111     G  G  R  S  D  K  D  Y  I  C  K  V  F  E  E  A  I  K  A  G  A  T  T
691    CTGGCCTGCCCGGACACGGTGGGGATCAACATGCCGCACGAATACGGGAAACTTGTGAGATACATCAAA
134     L  A  C  P  D  T  V  G  I  N  M  P  H  E  Y  G  K  L  V  R  Y  I  K
760    GCAAACACTCCTGGAATTGATGATGTTATCTTCAGCGCTCATTGTCACAATGACCTTGGTGTTGCTACC
157     A  N  T  P  G  I  D  D  V  I  F  S  A  H  C  H  N  D  L  G  V  A  T
829    GCCAACACAATCGCCGGTATATGTGCGGGAGCACGACAAGTCGAAGTAACAATCAATGGAATAGGTGAA
180     A  N  T  I  A  G  I  C  A  G  A  R  Q  V  E  V  T  I  N  G  I  G  E
898    AGAAGTGGGAATGCACCGCTTGAAGAGGTCGTGATGGCTTTGAAATGTCGAGGAGCATTTGTGATGGGT
203     R  S  G  N  A  P  L  E  E  V  V  M  A  L  K  C  R  G  A  F  V  M  G
967    GGTGTTTACACAAGAATAGACACACGCCAAATTATGGCTACCAGCAAGATGGTTCAAGAATATACTGGC
226     G  V  Y  T  R  I  D  T  R  Q  I  M  A  T  S  K  M  V  Q  E  Y  T  G
1036   TTGTATGTTCAGCCACATAAACCTATAGTTGGAGCCAACTGTTTTGTTCATGAGAGCGGCATTCACCAG
249     L  Y  V  Q  P  H  K  P  I  V  G  A  N  C  F  V  H  E  S  G  I  H  Q
1105   GATGGGATATTGAAAAATCGGAGTACATATGAGATCTTATCACCAGAAGATGTTGGAGTTGTAAAATCT
272     D  G  I  L  K  N  R  S  T  Y  E  I  L  S  P  E  D  V  G  V  V  K  S
1174   CAAAATTCAGGCATTGTTCTTGGAAAGCTTAGTGGACGTCATGCGGTTAAAGGTCGTCTGAAAGAGTTG
295     Q  N  S  G  I  V  L  G  K  L  S  G  R  H  A  V  K  G  R  L  K  E  L
1243   GGATATGGAATCAGTGATGAGAAACTGAACGAGGTTTTCTCACGGTTCAGAGATTTAACCAAGCAGAAA
318     G  Y  G  I  S  D  E  K  L  N  E  V  F  S  R  F  R  D  L  T  K  Q  K
1312   AAGAGAGTCACGGATGATGATCTAAGGGCATTAGTAACGTGTGGTGATGAAATCTTCTCCTCAGAGAAA
341     K  R  V  T  D  D  D  L  R  A  L  V  T  C  G  D  E  I  F  S  S  E  K
1381   TTAAACGGCACTAACGACAACGAAATCAACAGAAACGGCTATGTACCGTCTCCTCAGATTTCCTCTGTG
364     L  N  G  T  N  D  N  E  I  N  R  N  G  Y  V  P  S  P  Q  I  S  S  V
1450   GTATGAATTATGTGACCACGTCTGTTATGTTTGTGTATCACTGTAATATCAAGTTTCTTTTGAAACTTG
387     V  *
1519   TAATGAATAAAACAAGTTTCTTTTCCTATAAAAAAAAAAAAA
```

Figure S1. ***Cont.***

(c)

```
1       CTCTCTCTACTCACACACGCAAACATCATGACAATGATGATGAGCCTTACCACATCGTTACCATACCCT
1                                  M  T  M  M  M  S  L  T  T  S  L  P  Y  P
70      TTTCAAATCCTACTAGTCTTTAGCTTATCCATGGCATCAATCACTTTGCTTGGCCGAATACTATCAAGA
15       F  Q  I  L  L  V  F  S  L  S  M  A  S  I  T  L  L  G  R  I  L  S  R
139     CCCTCCAAAACCAAAAACCGGTCTCGTCAGCTTCCTCCCGGACCACCAGGATGGCCCATCCTAGGCAAT
38       P  S  K  T  K  N  R  S  R  Q  L  P  P  G  P  P  G  W  P  I  L  G  N
208     TTACCCGAACTAATGATGACTCGTCCTAGGCACAAATATGTCGGCATTGCCATGGAAGGGCAAAAACCG
61       L  P  E  L  M  M  T  R  P  R  H  K  Y  V  G  I  A  M  E  G  Q  K  P
277     GATATCGCATGTTTCAACTTCGCCGGAACACACGCCATCATCATAAACTCCGACGAGATCGCTCGAGAA
84       D  I  A  C  F  N  F  A  G  T  H  A  I  I  I  N  S  D  E  I  A  R  E
346     GCGCTTAAAGAGAGAGACGCCGACTTCGCAGACCGGCCTAATCTTTTCAACATGAGAACCATCGGAGGC
107      A  L  K  E  R  D  A  D  F  A  D  R  P  N  L  F  N  M  R  T  I  G  G
415     AATCACAAATCAATGGGGAACACACCCTACGGTGAACAGTTCATGAAGATGAAAAGAGTGATCAGAACG
130      N  H  K  S  M  G  N  T  P  Y  G  E  Q  F  M  K  M  K  R  V  I  R  T
484     GAGATTATGTCCGTTAAAACATTAAACATGTTAGAAGCTGCGAGAACTATTGAAGCGGACAACCTCCTT
153      E  I  M  S  V  K  T  L  N  M  L  E  A  A  R  T  I  E  A  D  N  L  L
553     GCTTACCTTCTCTCCATGTACAAACGGTCTGAGACTGCTGACGCTAGAGAGTTCTCGAGGGTTTATGGT
176      A  Y  L  L  S  M  Y  K  R  S  E  T  A  D  A  R  E  F  S  R  V  Y  G
622     TACGCTGTGACCATGAGATTGTTGTTTGGAAGGAGACATGTCACGAAAGAAAATGTTTTCTCCGACGAG
199      Y  A  V  T  M  R  L  L  F  G  R  R  H  V  T  K  E  N  V  F  S  D  E
691     GGAAGGTTAGGACAAGCGGAGAAAGATCATCTTGATGCGATTTTCGAAACCTTAAACTGTTTGCCGAGT
222      G  R  L  G  Q  A  E  K  D  H  L  D  A  I  F  E  T  L  N  C  L  P  S
760     TTCTCTCCGGCGGATTACTTGGAAAAATGGTTTAGAGGTTGGAACATTGATGGTCAAGAGGAGAGGGTG
245      F  S  P  A  D  Y  L  E  K  W  F  R  G  W  N  I  D  G  Q  E  E  R  V
829     GTAATGTCCTGTAATAAAGTTCGCAGTTACAACAATCCGATCATTGACGAGAGGGTGGAGTTATGGAGG
268      V  M  S  C  N  K  V  R  S  Y  N  N  P  I  I  D  E  R  V  E  L  W  R
898     GAAAAAGGTGGTAAAGCATCTGTTGAAGATTGGATTGATACTTTCATTACGCTAAAAGATGAAAATGGA
291      E  K  G  G  K  A  S  V  E  D  W  I  D  T  F  I  T  L  K  D  E  N  G
967     AAGTATTATATCACCCCGGATGAGGTCAAAGCTCAATGCGTTGAATTTTGTATAGCAGCGATCGATAAT
314      K  Y  Y  I  T  P  D  E  V  K  A  Q  C  V  E  F  C  I  A  A  I  D  N
1036    CCGGCAAATAACATGGAATGGACACTTGCGGAAATGTTAAAGAACCCGGAGATTCTCAAAAAAGCTTTG
337      P  A  N  N  M  E  W  T  L  A  E  M  L  K  N  P  E  I  L  K  K  A  L
1105    AAGGAGTTAGACGAAGTGGTGGGAAGAGACAGGCTTGTTCAGGAATCTGACATACCAAATCTAAACTAT
360      K  E  L  D  E  V  V  G  R  D  R  L  V  Q  E  S  D  I  P  N  L  N  Y
1174    TTAAAAGCTTGTTGCAGAGAAACATTCAGGATTCACCCTAGTGCTCATTATGTCCCTACACATGTGGCT
383      L  K  A  C  C  R  E  T  F  R  I  H  P  S  A  H  Y  V  P  T  H  V  A
1243    CGTCAAGATACCACCCTCGGGGGTTATTTCATTCCCAAAGGTAGCCACATTCATGTAGGCCGTCCTGGA
406      R  Q  D  T  T  L  G  G  Y  F  I  P  K  G  S  H  I  H  V  G  R  P  G
1312    ATAGGCCGGAGCTCCAAAATATGGACAGATCCATTGGTATACAAACCAGAGCGTCACCTAGAAGGAGAT
429      I  G  R  S  S  K  I  W  T  D  P  L  V  Y  K  P  E  R  H  L  E  G  D
1381    GGAATATCGAAGGAGCTTTCTCTGGTCGAAACTGAATTGCGTTTCGTGTCGTTTGGTACCGGTCGGCGT
452      G  I  S  K  E  L  S  L  V  E  T  E  L  R  F  V  S  F  G  T  G  R  R
1450    GGCTGCGTGGGTGTTAAAGTCGGGACGATCATGATGGTTATAATGTTGGCTAGGTTTCTTCAAGCGTTT
475      G  C  V  G  V  K  V  G  T  I  M  M  V  I  M  L  A  R  F  L  Q  A  F
1519    AATTGGAAACTCCATCCTGGTTATGGACCGTTAAGTCTAGAGGAGGATGATGCATTGCTTATGGCTAAG
498      N  W  K  L  H  P  G  Y  G  P  L  S  L  E  E  D  D  A  L  L  M  A  K
1588    CCTCTTCTCTTGTCCGTCGAGCCACGCTTGGCACCCAACCTTTATCCAAAATTTGCCCTTGAGTGAAAA
521      P  L  L  L  S  V  E  P  R  L  A  P  N  L  Y  P  K  F  A  L  E  *
1657    AAAAAGCACATTAACAGAAAAAGGAAACA
```

Figure S1. (**a**) Sequence analysis of nucleotide and deduced amino acid sequences of *BCAT4*; (**b**) Sequence analysis of nucleotide and deduced amino acid sequences of *MAM1*; (**c**) Sequence analysis of nucleotide and deduced amino acid sequences of *CYP79F1*. The start codon is indicated in purple. The stop codon is indicated in yellow. The BCAT beta family domain is indicated in green. The DRE TIM IPMS domain is indicated in blue. The p450 domain is indicated in red.

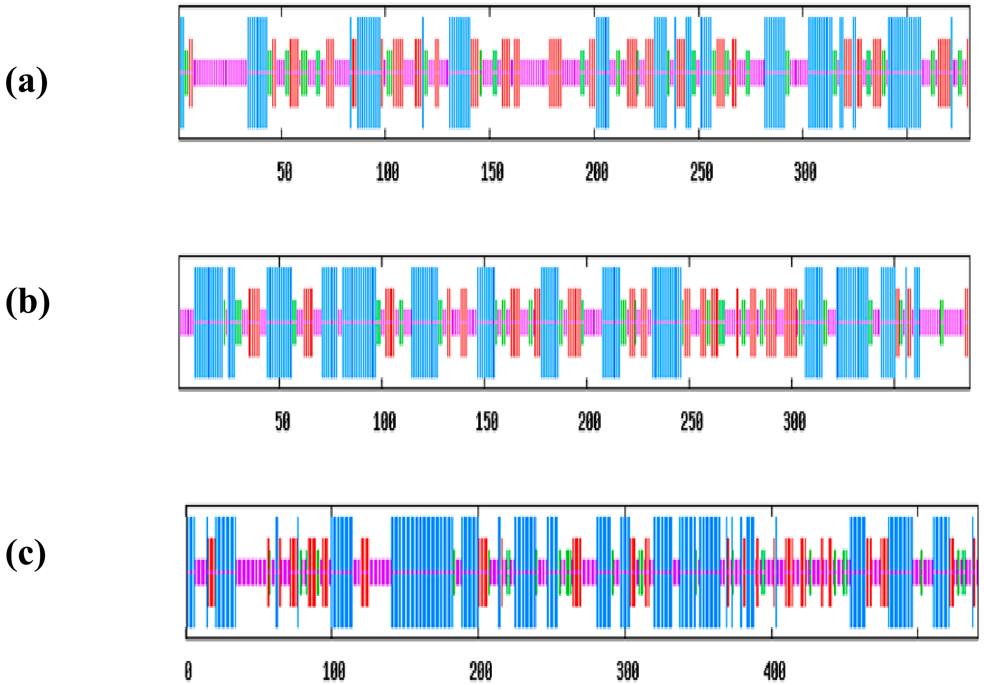

Figure S2. (**a**) The secondary structures of *BCAT4*; (**b**) The secondary structures of *MAM1*; (**c**) The secondary structures of *CYP79F1*. The longest vertical bar represents alpha helix. The second longest one represents extended strand. The third longest one represents the random coil. The shortest one represents beta turn.

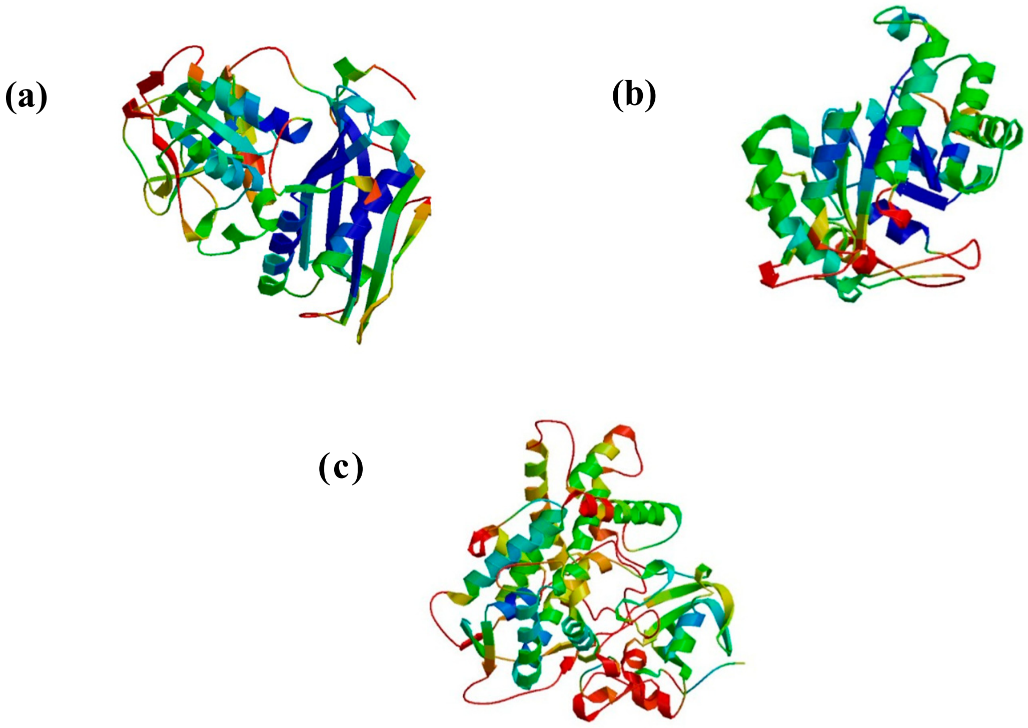

Figure S3. (**a**) Three-dimensional model structures of *BCAT4*; (**b**) Three-dimensional model structures of *MAM1*; (**c**) Three-dimensional model structures of *CYP79F1*.

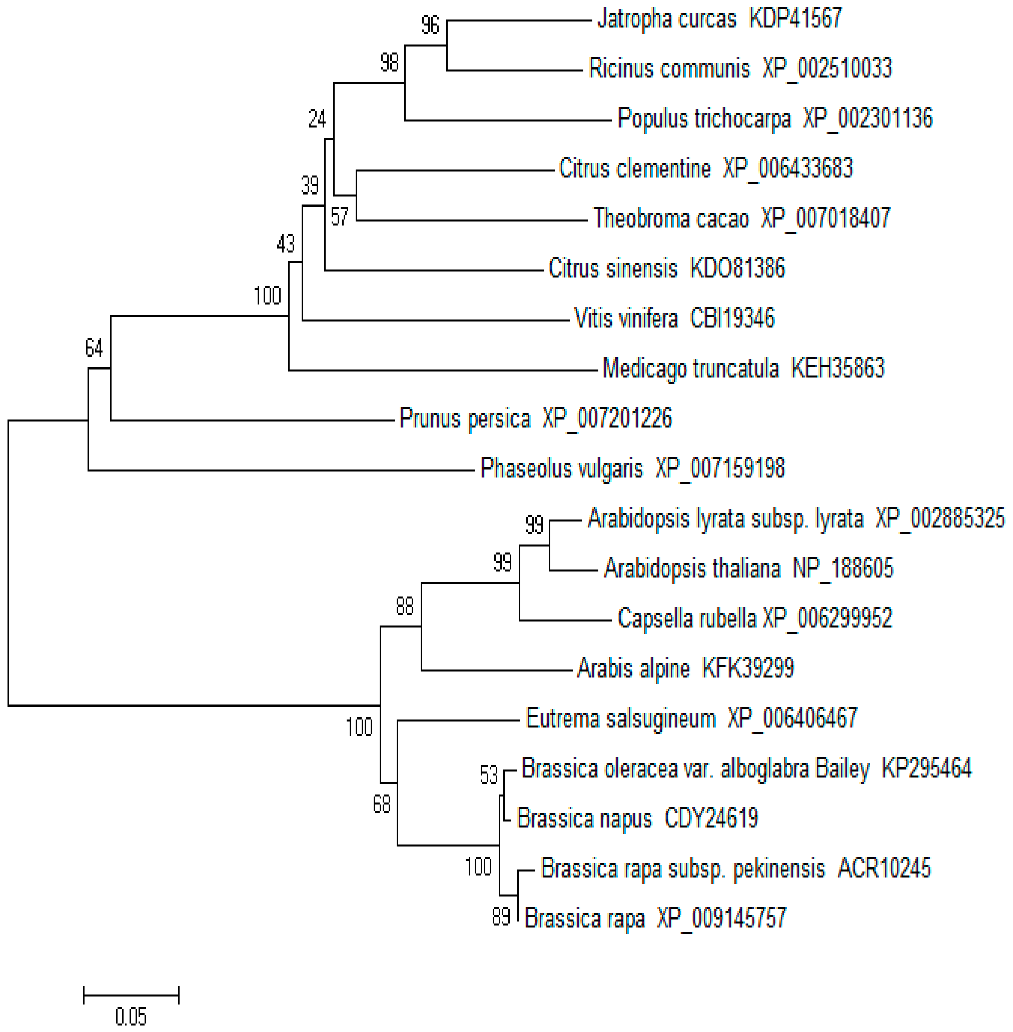

Figure S4. The phylogenetic trees of BCAT4 in Chinese kale with other plants. Accession numbers: *Arabis alpine* (KFK39299), *Arabidopsis thaliana* (NP_188605), *Arabidopsis lyrata* subsp. *lyrata* (XP_002885325), *Brassica napus* (CDY24619), *Brassica rapa* (XP_009145757), *Brassica rapa* subsp. *pekinensis* (ACR10245), *Capsella rubella* (XP_006299952), *Citrus clementine* (XP_006433683), *Citrus sinensis* (KDO81386), *Eutrema salsugineum* (XP_006406467), *Jatropha curcas* (KDP41567), *Medicago truncatula* (KEH35863), *Ricinus communis* (XP_002510033), *Phaseolus vulgaris* (XP_007159198), *Populus trichocarpa* (XP_002301136), *Prunus persica* (XP_007201226), *Theobroma cacao* (XP_007018407) and *Vitis vinifera* (CBI19346).

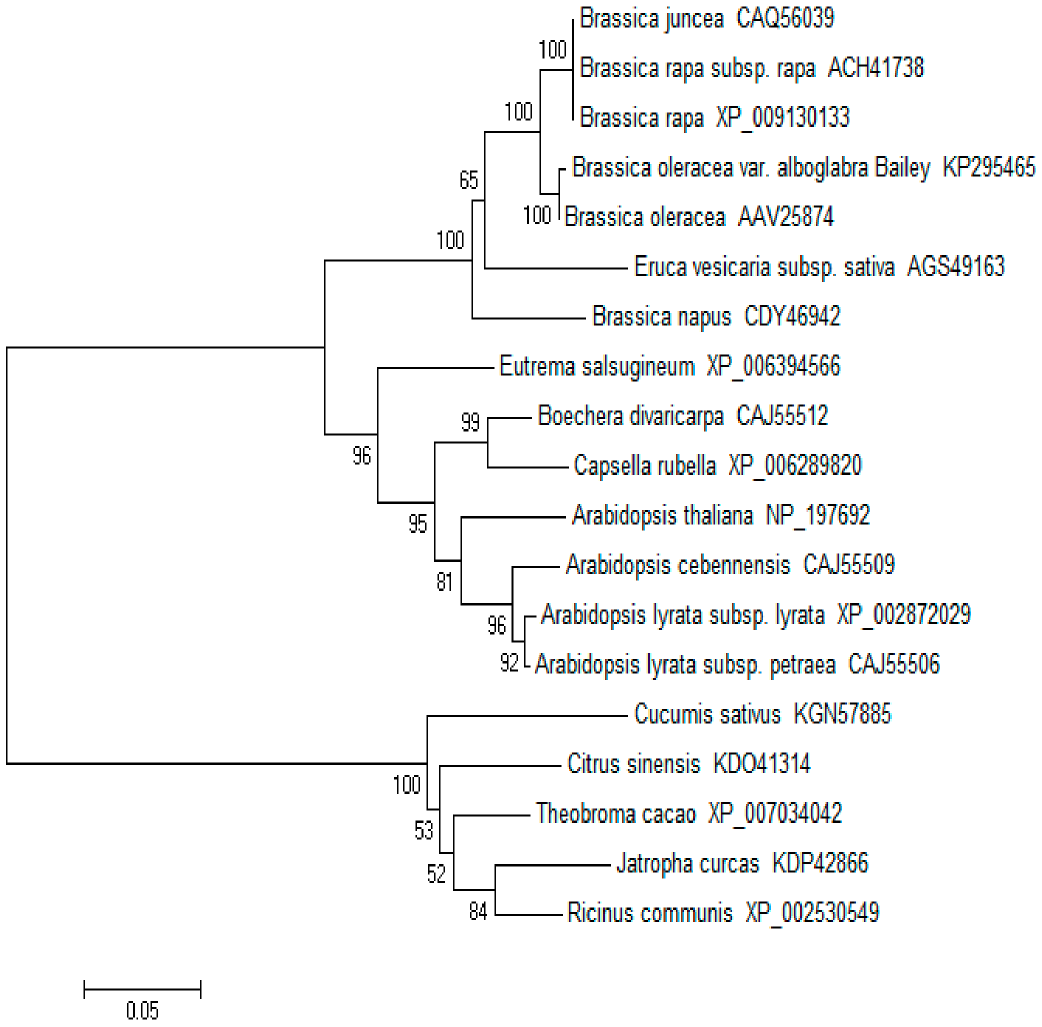

Figure S5. The phylogenetic trees of MAM1 in Chinese kale with other plants. Accession numbers: *Arabidopsis thaliana* (NP_197692), *Arabis alpine* (KFK27840), *Arabidopsis lyrata subsp. lyrata* (XP_002872029), *Arabidopsis cebennensis* (CAJ55509), *Arabidopsis lyrata* subsp. *petraea* (CAJ55506), *Brassica oleracea* (AAV25874), *Brassica juncea* (CAQ56039), *Brassica napus* (CDY46942), *Brassica rapa* (XP_009130133), *Brassica rapa* subsp. *rapa* (ACH41738), *Boechera divaricarpa* (CAJ55512), *Capsella rubella* (XP_006289820), *Cucumis sativus* (KGN57885), *Citrus sinensis* (KDO41314), *Eutrema salsugineum* (XP_006394566), *Eruca vesicaria* subsp. *sativa* (AGS49163), *Jatropha curcas* (KDP42866), *Ricinus communis* (XP_002530549) and *Theobroma cacao* (XP_007034042).

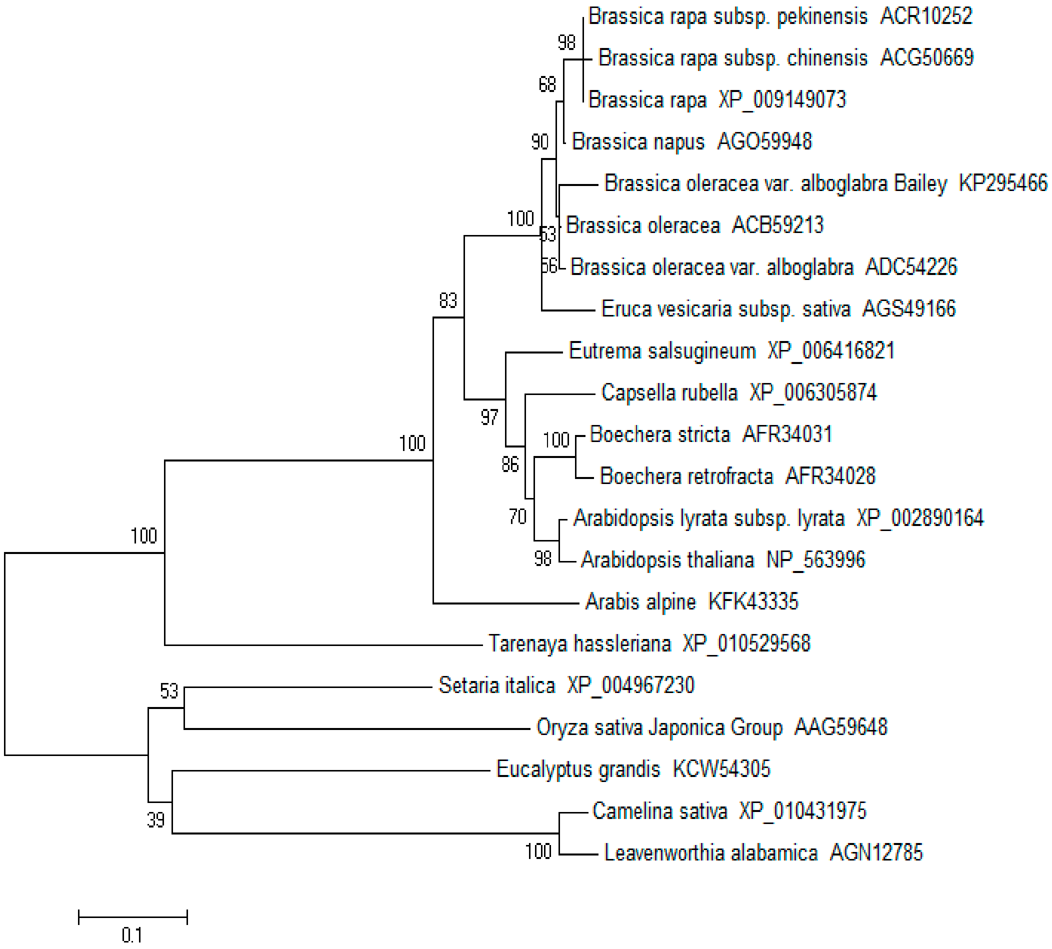

Figure S6. The phylogenetic trees of CYP79F1 in Chinese kale with other plants. Accession numbers: *Arabis alpine* (KFK43335), *Arabidopsis thaliana* (NP_563996), *Arabidopsis lyrata* subsp. *lyrata* (XP_002890164), *Brassica oleracea* var. *alboglabra* (ADC54226), *Brassica rapa* subsp. *pekinensis* (ACR10252), *Brassica rapa* (XP_009149073), *Brassica rapa* subsp. *chinensis* (ACG50669), *Brassica napus* (AGO59948), *Boechera retrofracta* (AFR34028), *Brassica oleracea* (ACB59213), *Boechera stricta* (AFR34031), *Camelina sativa* (XP_010431975), *Capsella rubella* (XP_006305874), *Eruca vesicaria* subsp. *sativa* (AGS49166), *Eutrema salsugineum* (XP_006416821), *Eucalyptus grandis* (KCW54305), *Leavenworthia alabamica* (AGN12785), *Setaria italica* (XP_004967230), *Oryza sativa Japonica Group* (AAG59648) and *Tarenaya hassleriana* (XP_010529568).
